# A simple and efficient total genomic DNA extraction method for individual zooplankton

**DOI:** 10.1186/s40064-016-3724-x

**Published:** 2016-12-01

**Authors:** Hanafiah Fazhan, Khor Waiho, Md. Sheriff Shahreza

**Affiliations:** Institute of Tropical Aquaculture, Universiti Malaysia Terengganu (UMT), 21030 Kuala Terengganu, Terengganu Malaysia

**Keywords:** TGDNA extraction, Copepod, PCR, Harpacticoid, PCR buffer, Vivantis

## Abstract

Molecular approaches are widely applied in species identification and taxonomic studies of minute zooplankton. One of the most focused zooplankton nowadays is from Subclass Copepoda. Accurate species identification of all life stages of the generally small sized copepods through molecular analysis is important, especially in taxonomic and systematic assessment of harpacticoid copepod populations and to understand their dynamics within the marine community. However, total genomic DNA (TGDNA) extraction from individual harpacticoid copepods can be problematic due to their small size and epibenthic behavior. In this research, six TGDNA extraction methods done on individual harpacticoid copepods were compared. The first new simple, feasible, efficient and consistent TGDNA extraction method was designed and compared with the commercial kit and modified available TGDNA extraction methods. The newly described TGDNA extraction method, “Incubation in PCR buffer” method, yielded good and consistent results based on the high success rate of PCR amplification (82%) compared to other methods. Coupled with its relatively consistent and economical method the “Incubation in PCR buffer” method is highly recommended in the TGDNA extraction of other minute zooplankton species.

## Background

Copepods are tiny multicellular organisms with size range between 0.1 and 1–2 mm that spread through different water bodies (Humes 1994; McKinnon et al. 2003; Pesce 2010). They even colonized harsh environment such as polar and hot spring water (Huys and Boxshall 1991). Taxonomic classification grouped copepod under Subphylum Crustacea due to the presence of two pairs of antennae, mandibles, maxillae on their heads and a pair of compound eyes (usually on stalks), a pair of appendages on each body segment for namely head, abdomen and thorax. The Subclass Copepoda is comprised of approximately 200 families, 1650 genera and 11,500 species (Humes 1994).

Currently, copepods are gaining attention in the aquaculture sector as live feed (Kahan et al. 1982; Kleppel and Hazzard 2002; Lee et al. 2005; Mckinnon et al. 2003; Peralta and Monica 2004; Watanabe and Kiron 1994; Williamson and Reid 2001). Classification of copepods in aquaculture farms is generally using conventional method based on morphological characters such as length of antenna, fifth walking leg and curvature coxa of the fifth pair of swimming legs (Huys and Boxshall 1991; Jagadeesan et al. 2009; Kabata 1979). An experienced taxonomist is needed in microscopic determination of copepods species and only copepods at late copepodite and adult stages only can be identified. The lack of precise and quantitative morphological characteristic analysis has made it difficult to classify due to their minute size (Böttger-Schnack and Machida 2011; Bucklin and Lajieunesse 1994; Dawson 2003; Huys and Boxshall 1991; Lindeque et al. 1999; McKinnon et al. 2003; Sneath and Sokal 1973; Suzuki et al. 2006; Weins 2000). Therefore, there is a need for a feasible, fast, reliable and precise technique in copepod species differentiation due to their abundances and morphological ambiguity. Molecular data such as DNA and RNA sequences provide complementary and informative data for systematic studies of copepods to determine their evolutionary relationship, taxonomy and even function of specific genes (Austin et al. 2016; Bucklin et al. 1999, 2000; Burton et al. 2007; Chow et al. 2008; Dennis et al. 2009; Lindeque et al. 1999; Machida and Tsuda 2010; Palumbi and Benzie 1991; Rhee et al. 2009; Song et al. 2008; Suzuki et al. 2006; Thum and Derry 2008; Thum and Harrison 2008).

Extraction of total genomic DNA is one of the primary steps before proceeding onto subsequent molecular studies. The total genomic DNA (TGDNA) extraction of individual larger animals and plants are easier by using conventional method and various commercial kits. But, the TGDNA extraction for the individual tiny organism (<1 mm) such as copepods proves to be difficult as they have comparatively lesser amount of target DNA to start with (Saiki et al. 1988). Schizas et al. (1997) mentioned that skills are needed in handling copepods during TGDNA extraction because copepods especially from the order Harpacticoida (epibenthic) live in close contact with sediment, fungi, bacteria and other zooplanktons. It is important to use individual copepod rather than a clump or a population for TGDNA extraction in genetics research to avoid contamination or mixed species. Various TGDNA extraction techniques (conventional methods, modified methods and commercial extraction kits) were compared in this research in order to identify a feasible, efficient and consistent TGDNA extraction method. In this study, a simple method that uses less chemicals and minimal handling of the sample while being capable of producing consistent positive results (validated by successful polymerase chain reaction (PCR) amplification) for extracting the TGDNA from individual harpacticoid copepod has been developed.

## Methods

### Experimental organism

Live samples of harpaticoid copepod, *Leptocaris canariensis* were collected from pure culture maintained in laboratory of Universiti Malaysia Terengganu, Terengganu Malaysia. The use of copepods and their extraction methods in this research were approved by the Institute of Tropical Aquaculture, Universiti Malaysia Terengganu.

### Comparison of TGDNA extraction methods

TGDNA of *L. canariensis* was extracted using six different methods, namely CTAB DNA extraction method that was modified from the method established by Winnepenninckx et al. (1993), modified phenol chloroform DNA extraction method from Pearson and Stirling (2003), KAPA Express Extract kit (KAPA Biosystems Inc, USA), Direct Boiling method designed by Vestheim et al. (2005), TGDNA extraction using “Incubation in lysis buffer and proteinase K” by Burton et al. (2007) and TGDNA extraction using “Incubation in PCR buffer”. The detection methods of the availability of TGDNA were done by using Agarose Gel Electrophoresis, spectrophotometer and PCR. 50 *L*. *canariensis* individuals were used for each TGDNA extraction methods. The efficiency and consistency of the methods used were based on the success in DNA extraction detected by agarose gel electrophoresis (AGE), spectrophotometer or PCR.

#### Modified CTAB

Individual *L*. *canariensis* was minced under dissecting microscope in 50 μL 2× CTAB buffer (2% w/v CTAB, 1.4 M NaCl, 0.2% v/v 2-mercaptoethanol, 20 mM EDTA, 100 mM Tris–HCl (pH 8.0), 0.1 mg/mL proteinase K) using a fine needle. The minced sample was transferred into a 1.5 mL microcentrifuge tube containing premixed 100 μL of 2× CTAB buffer with 5 μL Proteinase K (20 mg/mL) and incubated at 60 °C for 1–3 h. The incubated sample was then mixed with 60 μL Chloroform: Isoamyl alcohol (24:1) by shaking the mixture for 2 min and then centrifuged at 13,000 rpm for 10 min. Supernatant was carefully transferred into a new 1.5 mL microcentrifuge tube before repeating previous steps of adding C:IA and then centrifuged. Supernatant was transferred into a new 1.5 mL microcentrifuge tube, mixed with 60 μL of absolute ethanol and centrifuged (13,000 rpm/10 min). The supernatant was discarded. The pellet formed was washed twice by adding 50 μL of 70% ethanol and centrifuged (13,000 rpm/10 min). Pellet was air dried for about 1 h and dissolved in 50 μL sterile double distilled water (ddH_2_O).

#### Modified phenol chloroform

An individual *L*. *canariensis* was minced under dissecting microscope in 50 μL lysis buffer [10 mM NaCl, 20 mM Tris–HCl, pH 8.0, 1 mM EDTA, 1% sodium dodecyl sulfate (SDS)] using a fine needle. The minced sample was transferred into a 1.5 mL microcentrifuge tube containing premixed 60 μL of lysis buffer with 5 μL Proteinase K (20 mg/mL) and incubated at 60 °C for 1–3 h. The incubated sample was then mixed with 60 μL Phenol: Chloroform (1:1) by shaking the mixture for 2 min and centrifuged at 13,000 rpm for 10 min. The remaining supernatant was transferred into a new 1.5 mL microcentrifuge tube. 60 μL of Phenol: Chloroform: Isoamyl alcohol (25:24:1) was added and the mixture was centrifuged (13,000 rpm/10 min). Supernatant was transferred into a new 1.5 mL microcentrifuge tube, mixed with 60 μL of absolute ethanol and centrifuged (13,000 rpm/10 min). The supernatant was discarded and the pellet formed was washed twice by adding 50 μL of 70% ethanol and centrifuged (13,000 rpm/10 min). Pellet was air dried for about 1 h and dissolved in 50 μL sterile ddH_2_O.

#### KAPA Express Extract kit

KAPA Express Extract kit was used based on the procedure provided by the manufacturer on fish tissue extraction. An individual *L*. *canariensis* was minced under dissecting microscope in 10 μL sterile ddH_2_O using a fine needle. The minced mixture was transferred into a 0.2 mL PCR tube and 100 μL lysis solutions were added. PCR tube was sealed and transferred into BIORAD MyCycler™ thermal cycler machine and incubated (60 °C/10 min and 95 °C/5 min). The sample was centrifuged (14,000 rpm/60 s) and the supernatant was transferred into a new PCR tube.

#### Direct boiling

An individual *L*. *canariensis* was minced under dissecting microscope in 10 μL sterile ddH_2_O using fine needle. The minced sample was transferred into a 0.2 mL PCR tube containing 30 μL ddH_2_O and boiled at 100 °C for 5 min.

#### Incubation in lysis buffer and proteinase K

An individual *L*. *canariensis* was minced under dissecting microscope in 10 μL sterile ddH_2_O using a fine needle. The minced sample was transferred into a 1.5 mL microcentrifuge tube containing 40 μL of lysis buffer (10 mM NaCl, 20 mM Tris–HCl, pH 8.0, 1 mM EDTA, 1% SDS), 10 μL Proteinase K (20 mg/mL) and incubated at 60 °C for 60 min. The incubated sample was then mixed with 60 μL of cold absolute ethanol for 30 min in 4 °C and centrifuged (13,000 rpm/15 min). The supernatant was discarded and the pellet formed was washed by 100 μL of 70% ethanol was added and centrifuged (13,000 rpm/10 min) twice. The pellet was then air dried for about 1 h and dissolved in 50 μL sterile ddH_2_O.

#### Incubation in PCR buffer

The PCR buffer used in this study was PCR buffer A (500 mM KCl, 100 mM Tris–HCl and 0.1% Triton™X-100) (VIVANTIS Technologies, MY). An individual *L*. *canariensis* was minced under dissecting microscope in 5 μL sterile ddH_2_O using a fine needle. The minced mixture was transferred into a 0.2 mL PCR tube containing premixed 5 μL of sterile ddH_2_O and 2.5 μL VIVANTIS PCR buffer A. The mixture was incubated for 15 min at room temperature. The availability of TGDNA was only assessed by PCR.

### Detection of TGDNA

#### AGE

A 1% agarose gel [1× Tris-acetate-EDTA (TAE) buffer pre-stained with ethidium bromide] was prepared. The samples were run at 70 V for 45 min.

#### Spectrophotometer

Optical density (OD) readings at 260 and 280 nm of TGDNA extraction products were carried out to determine the availability, quantity and quality of obtained DNA using spectrophotometer.

#### PCR

Amplifications were done in a thermal cycler (BIO-RAD MyCycler™). The primers used in were ITS1a and ITS1r designed by Dennis et al. (2009) and LCO-1490 and HCO-2198 design by Folmer et al. (1994). The PCR reactions carried out with 40 cycles of a 25 μL reaction volume containing 5.6 μL mixture of sterile ddH_2_O, 2.5 μL of 10× PCR buffer, 1.0 μL of dNTP (10 mM each), 1.5 μL of each primer (2.5 µM), 12.5 μL of DNA template and 0.4 μL of *Taq* Polymerase manufactured by Vivantis Technologies Sdn. Bhd. (5 μ/μL). The thermal cycle profile was as follows: denaturation at 94 °C for 45 s, annealing at 52 °C for 45 s (for both primers), and extension at 72 °C for 60 s. The PCR products were run on a 1% AGE pre-stained with ethidium bromide for band characterization using ultraviolet trans-illumination. The negative control was prepared without the DNA template in PCR mixture and provided in each amplification reaction.

## Results

The detection of TGDNA of an individual *L*. *canariensis* was not applicable by using AGE and spectrophotometer due to the minute concentration of extracted TGDNA. Out of the three detection methods, only PCR was the most practical approach to detect the extracted TGDNA from individual *L*. *canariensis* (Table 1).

**Table 1:** Detection methods and success rate (efficiency)

TGDNA extraction methods	Detection methods			Success rate (efficiency) (%)
	AGE	Spectrophotometer	PCR	$\frac{success}{50\;trails} \times 100\%$
2× CTAB	−	−	−	0
Phenol chloroform	−	−	−	0
KAPA express extract	−	−	+	46
Direct boiling	−	−	−	0
Incubation in lysis buffer and proteinase K	−	−	+	18
Incubation in PCR buffer	−	−	+	82

+ = positive; − = negative

**Fig. 1:**
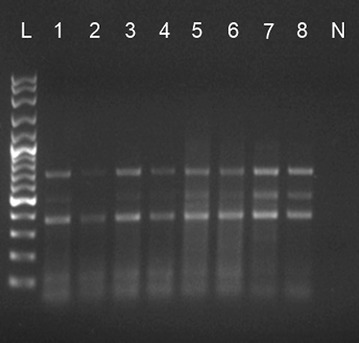
AGE photograph showing partial ITS1 gene bands of *L. canariensis* extracted using “incubation in PCR Buffer” method. AGE was run using 1% TAE agarose gel at 70 V for 45 min. *L* 100 kb Ladder; *L1–L8* PCR product from samples 1–8; *N* negative control

**Fig. 2:**
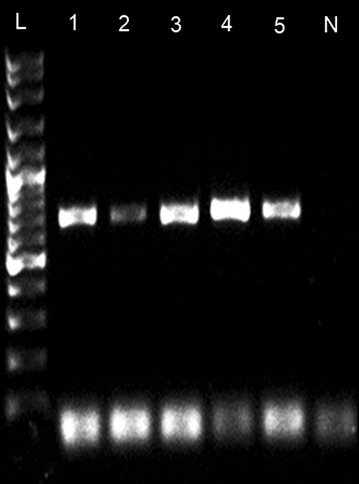
AGE photograph showing partial COI gene band of *L. canariensis canariensis* extracted using “incubation in PCR Buffer” method. AGE was run using 1% TAE agarose gel at 70 V for 45 min. *L* 100 kb Ladder; *L1–L5* PCR product from sample 1–5; *N* negative control

Three out of six methods successfully extracted TGDNA from individual copepod, *L*. *canariensis*, as indicated by the presence of PCR products. Comparatively, “Incubation in PCR buffer” method was the most feasible, consistent and efficient method with the highest success rate (82%) (Figs. 1, 2), followed by KAPA Express Extract Kit (46%) and “Incubation in lysis buffer and proteinase K” method (18%) (Table 1). The “Incubation in PCR buffer” method successfully and consistently amplifies the partial ITS1 gene of nuclear DNA and partial COI gene of mitochondrial DNA. This indicated that this method was able to extract nuclear and mitochondrial genome itself. Direct sequencing of the partial COI gene of *L*. *canariensis* of this study showed 77% similarities with partial mitochondrial COI gene region of calanoid copepods, *Boeckella brasiliensis*. The mitochondrial COI sequence was submitted to GenBank, NCBI, with Accession Number JF707331 (Waiho et al. 2013).

## Discussion

The amplified TGDNA product, even in initial low concentration, gave positive result in PCR reaction as expected (Grunenwald 2003; Raven and Johnson 2002). The best concentration of TGDNA required to yield satisfying results in PCR is between 100 and 500 ng, although concentration as low as 50 ng was reported to yield positive results as well (Creighton 1999). In this study, the observable PCR product bands in gel electrophoresis showed that the TGDNA from *L. canariensis* was successfully extracted although the initial TGDNA concentration from a single copepod is too low to be quantified via AGE or spectrophotometer. The applied methods to detect the presence of DNA in present study, i.e. spectrophotometer, gel electrophoresis and PCR are common DNA detection methods used by most researchers (Chen et al. 2010; de Oliveira et al. 2014; Wang and Wang 2012; Yoganandhan et al. 2003). In addition, due to the high sensitivity of PCR compared to the other two DNA detection methods, researchers also have resorted to use only PCR to detect the presence of DNA and directly sequence the targeted gene (Dashti et al. 2009; Englen and Kelley 2000; Vasuki et al. 2001). Currently available DNA extraction kits such as DNAMITE DIRECT DNA extraction kit (Microzone Ltd., UK), MightyPrep reagent for DNA (Takara Bio Company, USA), Phire animal tissue direct PCR kit (Thermo Fisher Scientific Inc, USA) and Extract-N-Amp tissue PCR kit (Sigma-Aldrich Co., Germany) also focused on the sensitivity of PCR and the inconsistency of spectrophotometer and gel electrophoresis, and directly skip DNA quantification steps prior PCR in their kit manual (extracted DNA are PCR-ready), thereby saving time and cost.

The ability to extract TGDNA using Vivantis PCR buffer A is attributed to the components incorporated in it. The Vivantis PCR buffer A contains 500 mM KCl, 100 mM Tris–HCl (pH 9.1 at 20 °C) and 0.1% Triton™X-100. According to Sha et al. (2008), Triton™X-100 is normally used as a detergent in lysis buffer and has the ability to denature membrane protein of the cell, resulting in the release of cell components including TGDNA. Combination of detergent and salts was reported to increase DNA extraction ability in cells as salts such as KCl provides a hypotonic environment that promotes cell lysis (Raven and Johnson 2002). Apart from 10× buffer A used in this study, almost all commercial PCR buffers contain detergent and salt as well that are essential in the lysis of cell membrane (i.e. KCl, Tris–HCl, Triton™X-100 and ammonium sulfate).

The primary advantages of TGDNA extraction using “Incubation in PCR buffer” method over other extraction methods used in this study are simplicity and consistency. This method is also relatively cheaper in comparison to the other two methods (KAPA Express Extract Kit and “Incubation in lysis buffer and proteinase K” method) that were able to yield positive PCR results. “Incubation in PCR buffer” method involves only a few simple and short procedures, thereby minimizing potential exposure time of DNA to other contaminants and risk of being degraded. Mishandling or improper pipetting technique can be avoided as the reaction involves only one chemical (buffer A) during extraction. In addition, the DNA reduction can be avoided as well because due to the limited TGDNA obtainable in copepod, conventional TGDNA extraction methods with longer procedures and more chemicals involved will tend to reduce the quantity and quality of TGDNA along with their extraction steps. The overnight incubation at 4 °C done in this study was proven to be able to extract sufficient amount of DNA for subsequent PCR amplification. Long incubation period was needed for chemicals in PCR buffer A to react and lyse cells, releasing TGDNA as no proteinase K was applied. Previous research done on copepods used PCR buffers for brief pre-incubation, before subjecting copepod samples to commercial DNA extraction kit (GeneReleaser™) containing proteinase K (Easton et al. 2010; Schizas et al. 1997), “Incubation in PCR buffer” method avoids the need to use either proteinase K or expensive commercial DNA extraction kit, thereby saving cost yet did not compromise the outcome.

The “Incubation in lysis buffer and proteinase K” method produced inconsistent results and is not recommended to be used in the TGDNA extraction of copepods. On the other hand, the KAPA Express Extract kit can be considered as an alternative method to extract the TGDNA of copepods, as it is simple in procedure and can yield at least 42% success rate in PCR amplification.

## Conclusions

We report in this study a simple, feasible, efficient and consistent TGDNA extraction method, i.e. “Incubation in PCR buffer” method, to extract TGDNA from individual zooplankton. This study also shows that when only a single zooplankton was used, the TGDNA extracted was undetected using normal TGDNA detection methods (i.e. AGE and spectrophotometer) due to their very low concentration. The “Incubation in PCR buffer” method described in this study is highly applicable in future research on the molecular aspects of zooplankton such as molecular identification or population ecological studies.

## Abbreviations

AGE: agarose gel electrophoresis
ddH_2_O: double distilled water
min: minute
PCR: polymerase chain reaction
SDS: sodium dodecyl sulfate
TAE: Tris-acetate-EDTA
TGDNA: total genomic DNA
